# Network-based functional enrichment

**DOI:** 10.1186/1471-2105-12-S13-S14

**Published:** 2011-11-30

**Authors:** Christopher L Poirel, Clifford C Owens, Murali

**Affiliations:** 1 Department of Computer Science, Virginia Tech, Blacksburg, VA, USA; 2 ICTAS Centre for Systems Biology of Engineered Tissues, Virginia Tech, Blacksburg, VA, USA

## Abstract

**Background:**

Many methods have been developed to infer and reason about molecular interaction networks. These approaches often yield networks with hundreds or thousands of nodes and up to an order of magnitude more edges. It is often desirable to summarize the biological information in such networks. A very common approach is to use gene function enrichment analysis for this task. A major drawback of this method is that it ignores information about the edges in the network being analyzed, i.e., it treats the network simply as a set of genes. In this paper, we introduce a novel method for functional enrichment that explicitly takes network interactions into account.

**Results:**

Our approach naturally generalizes Fisher’s exact test, a gene set-based technique. Given a function of interest, we compute the subgraph of the network induced by genes annotated to this function. We use the sequence of sizes of the connected components of this sub-network to estimate its connectivity. We estimate the statistical significance of the connectivity empirically by a permutation test. We present three applications of our method: i) determine which functions are enriched in a given network, ii) given a network and an interesting sub-network of genes within that network, determine which functions are enriched in the sub-network, and iii) given two networks, determine the functions for which the connectivity improves when we merge the second network into the first. Through these applications, we show that our approach is a natural alternative to network clustering algorithms.

**Conclusions:**

We presented a novel approach to functional enrichment that takes into account the pairwise relationships among genes annotated by a particular function. Each of the three applications discovers highly relevant functions. We used our methods to study biological data from three different organisms. Our results demonstrate the wide applicability of our methods. Our algorithms are implemented in C++ and are freely available under the GNU General Public License at our supplementary website. Additionally, all our input data and results are available at http://bioinformatics.cs.vt.edu/~murali/supplements/2011-incob-nbe/.

## Background

The functioning of a living cell is governed by an intricate network of interactions among different types of molecules. These interactions transduce external signals, control gene expression, protein synthesis and localization, chemically modify protein activities, and drive metabolic and biochemical reactions. Considerable effort in molecular and cellular biology has been expended over the last 50 years by individual research groups on testing and detecting interactions on a small scale. The results of these experiments are enshrined in the literature. In the last few years, a number of efforts have manually curated the literature and created databases of these interactions [1-3]. More recently, the genomic revolution has inspired the development of experimental technologies that can detect interaction networks in a high-throughput manner and on a genome-wide scale. For example, the yeast 2-hybrid screen has been scaled up to unveil protein-protein interaction networks containing tens of thousands of interactions in a number of organisms [4,5]. In a similar vein, the chromatin immunoprecipitation on a microarray (ChIP-on-chip) technology allows the detection of the targets of a specified transcription factor on a genome-wide scale [6]. These developments have made molecular interaction networks pervasive in systems biology.

Concomitantly, a number of computational approaches have been developed to analyze networks and their properties. Foremost among them are methods to reverse engineer gene regulatory networks by integrating gene expression data with other types of ’omic data [7]. Such interactions usually relate the expression of a gene to that of other genes in the cell [8]. Another broad class of methods overlay gene expression data for a condition on the wiring diagram to compute the cell’s response network for that condition [9-11]. Networks of these types can contain hundreds or thousands of nodes and an order of magnitude more edges. For example, the B-cell interactome [12,13], a network of experimentally verified or computationally predicted protein-DNA, protein-protein, and transcription factor-modulator interactions, contains nearly 6000 nodes and over 64,000 edges. It is often desirable to summarize the biological information in such networks. A very common approach is to perform enrichment analysis of the terms in some catalog such as the Gene Ontology [14-16]. Enriched functions and processes are very useful in summarizing the main biological themes of a large network at a high level, as a preliminary to more detailed mechanistic studies. When applied to reverse-engineered or response networks, a major drawback of functional enrichment is that it ignores information about the edges in the network being analyzed, i.e., it treats the network simply as a set of genes. Therefore, a function may appear to be enriched in a network, but the genes annotated with that function may be highly disconnected within the network. In such cases, it is difficult to interpret the relevance of that function to the network.

In this paper, we introduce a novel method for functional enrichment that explicitly takes network interactions into account. Our approach naturally generalizes Fisher’s exact test, a widely-used gene set-based technique. We use the sequence of sizes of the connected components of the network to estimate its degree of connectivity. We estimate the statistical significance of this connectivity empirically by a permutation test.

It may be argued that one approach that mitigates the drawback of using gene set enrichment on networks is to find clusters within the network and then compute enriched functions within them. Since clusters are usually densely-connected, enriched functions are likely to induce connected subgraphs within clusters. Our approach is distinct from finding enriched functions in clusters. Clustering algorithms typically compute dense subgraphs. In contrast, we detect subgraphs (defined by genes annotated with specific functions) that are more connected than may be expected at random. In our results, we show examples of subgraphs that clustering algorithms may not detect.

We showcase three applications of our approach: *i*) determine which functions are enriched in a reverse-engineered network, *ii*) given a network and a response sub-network of genes within that network, determine which functions are enriched in the sub-network, and *iii*) given two experimentally derived networks, determine the functions for which the connectivity improves when we merge the second network into the first. These analyses use data from three different species (human, rat, and baker’s yeast). They demonstrate that our conceptualization of network-based functional enrichment is a powerful framework for addressing a diverse variety of interesting biological questions about molecular interaction networks.

### Formulation of functional enrichment

**Figure 1:**
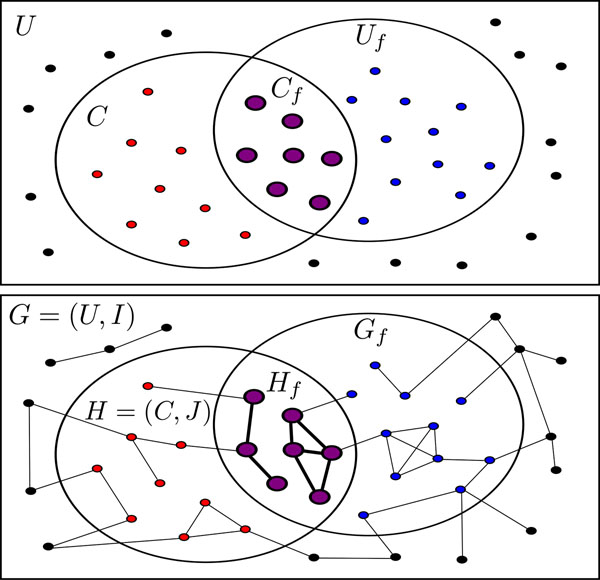
**Functional enrichment cartoons**. (Top) The standard formulation of functional enrichment computes the statistical significance of the size of *C_f_*, the overlap between an interesting collection *C* of genes and the set *U_f_* of genes annotated with function *f*. (Bottom) Our network-based approach to functional enrichment computes the statistical significance of the connectivity of *H_f_*, the network induced by the intersection of the interesting collection *C* and the set *U_f_* of genes annotated by the function.

The problem of functional enrichment is usually formulated as follows. We have a universe *U* of genes and an “interesting collection” *C* ⊂ *U* of genes. We desire to evaluate the functional coherence of *C* based on the annotations of the genes in it. To this end, we have access to a set *F* of biological functions. For each function *f* ∈ *F*, let *U_f_* ⊆ *U* denote the set of genes annotated by *f*. Furthermore, let *C_f_* = *C* ∩ *U_f_* denote the subset of genes in *C* that are annotated by *f*. We will use lowercase letters to denote the cardinalities of the corresponding sets, which are named by uppercase letters. See Figure 1 (top) for an illustration. The standard formulation of functional enrichment in terms of the one-sided version of Fisher’s exact test asks the following question:

If we select a set *X* of *u_f_* genes uniformly at random (without replacement) from the set of all genes *U*, what is the probability that *X* ∩ *C* will contain *c_f_* or more genes?

Thus, we are interested in the probability that a random set of *u_f_* genes would contain *c_f_* or more genes from *C*. As is well known, we can compute this probability by:(1)

Now let us consider the setting in which network-based functional enrichment is relevant. We are given an undirected graph *G* = (*U*, *I*), where *U* is the set of all genes (as before) and *I* is a set of edges. We are also given a subgraph *H* = (*C*, *J*) of *G*, where *C* (the “interesting collection” of genes) is a subset of *U*, and *J* is a subset of *I*. Note that *H* may not be the subgraph of *G* induced by *C*, i.e., some edges of *I* that connect node pairs in *C* may be missing from *J*. Given a function *f*, define *H_f_* to be the subgraph of *H* induced by the genes in *U_f_* ∩ *C*, i.e., the subgraph of *H* induced by the genes that are annotated by *f*. We desire to use statistics of *H_f_* to estimate whether the function *f* is enriched in *H* or not. This concept is illustrated in Figure 1 (bottom). Intuitively, even if *H_f_* is highly disconnected, existing methods may declare *f* to be highly enriched in *H*. However, it is difficult to interpret the biological relevance of such a function. Therefore, we would like to incorporate the connectedness of *H_f_* into its evaluation.

Ideally, *f* is highly statistically significant if *H_f_* contains only one connected component, and *f* is statistically insignificant if *H_f_* contains only singletons (or many small components). We define the *size* of a connected component as the number of nodes in that component. Suppose *H_f_* has *m* connected components whose sizes are *a*_1_, *a*_2_,…, *a_m_*, where *a_i_* ≥ *a_i_*_+1_, 1 ≤ *i* <*m*. Using the abbreviation *“cs”* for “component sizes,” we will use *cs*(*H_f_*) to denote this non-increasing sequence of numbers. We would like to estimate the statistical significance of *f* in terms of *cs*(*H_f_*). If *X* is a subset of *U*, we abuse notation and use *H_X_* to denote the subgraph of *H* induced by *X*. Drawing a parallel with the formulation of functional enrichment in the network-free case, we pose the following question:

If we select a set *X* of *u_f_* genes uniformly at random (without replacement) from the set of all genes *U*, what is the probability that *cs*(*H_X_*) ≥ *cs*(*H_f_*)?

Clearly, answering these questions requires that we define how we can compare different values of *cs*(), i.e., decide if one sorted sequence of connected component sizes is “greater than” another. Note that two distinct subsets *X* and *Y* of *U* may induce different subgraphs of *H* (i.e., *H_X_* ≠ *H_Y_*) that have the same sequence of component sizes. Conversely, *H_X_* and *H_Y_* may have the same number of nodes, yet *cs*(*H_X_*) and *cs*(*H_Y_*) may be vastly different. (Consider the case where |*X* ∩ *C*| = |*Y* ∩ *C*|, and *H_X_* is a clique while *H_Y_* is a collection of singletons.) It appears difficult to determine the null distribution of *cs*(*H_X_*) analytically. Thus, we developed two sampling-based approaches, discussed in “Methods”, to compute the statistical significance of *cs*(*H_f_*) empirically.

## Methods

As before, let *G* = (*U*, *I*) be a network whose nodes are the universal set of genes and let *H* = (*C*, *J*) be a network whose nodes are the interesting collection of genes. We sometimes refer to *G* and *H* as the *universal network* and the *interesting network*, respectively. Let *H_f_* be the subgraph of *H* induced by the nodes annotated with function *f*.

First, we elaborate on our method for comparing different values of *cs*(), i.e., sorted lists of network component sizes. Next, with a method to compare subnetworks in hand, we proceed to compute the statistical significance of *cs*(*H_f_*) empirically using two sampling-based approaches.

### Comparing sequences of component sizes

Let *A* = {*a*_1_, *a*_2_,…, *a_m_*} and *B* = {*b*_1_, *b*_2_, …, *b_n_*} be sorted sequences such that *a_i_* ≥ *a_i_*_+1_ for 1 ≤ *i* <*m*, and *b_i_* ≥ *b_i_*_+1_ for 1 ≤ *i* <*n*. Since the *a_i_*’s and *b_i_*’s represent component sizes, we assume all values are positive. If *m* <*n*, we pad *A* with zeros by setting *a_i_* = 0 for *m* <*i* ≤ *n*. We pad *B* similarly in the case that *n* <*m*.

Now, we naturally define *A* = *B* if and only if *a_i_* = *b_i_* for all 1 ≤ *i* ≤ max(*m*, *n*). Otherwise, we define *A* <*B* if and only if there exists some index *i*, 1 ≤ *i* ≤ max(*m*, *n*), such that *a_i_* <*b_i_*, and *a_j_* = *b_j_* for all *j* <*i*. If neither of these cases hold, we say that *B* <*A*. Essentially, we walk along *A* and *B* simultaneously (which have the same length after padding) until we find an index *i* where *a_i_* ≠ *b_i_*. The smaller sequence is the one that contains the smaller of those two values.

### Function randomization

The function randomization approach for computing the statistical significance of *cs*(*H_f_*) parallels the sampling-based alternative to the analytical solution for the one-sided version of Fisher’s exact test (Equation 1). In the sampling-based solution for Fisher’s exact test (that is not network-based), we repeatedly select a set *X* of *u_f_* genes uniformly at random from the universe of genes *U*. We then calculate an empirical *p-*value for the function *f*, which represents the statistical siginificance of the size of *C_f_*, as the fraction of samples for which |*X* ∩ *C*| ≥ *c_f_*, i.e., the size of the intersection between the randomly selected set *X* and the interesting collection of genes is at least as large as *C_f_*. If we repeat this process many times, the empirical value converges to the analytical value computed by Equation 1.

In the case of network-based enrichment, we have not been able to derive the null distribution of *cs*(*H_X_*) analytically. Therefore, we apply a sampling-based algorithm similar to the one used in the non-network case. We repeatedly select a set *X* of *u_f_* genes uniformly at random from the universal genes *U*. At each iteration we compute the subgraph of *H* = (*C*, *J*) induced by genes in *X* ∩ *C* and call this subgraph *H_X_*. We calculate the *p-*value for *f* as the fraction of random choices of *X* for which *cs*(*H_X_*) ≥ *cs*(*H_f_*). This *p*-value is an empirical estimate of the probability that the intersection between the interesting network and a randomly-selected subgraph of the universal network is more well-connected than *H_f_*.

This network-based formulation of functional enrichment strongly parallels the traditional non-network formulation. In fact, notice that if we remove all edges from *G* and from *H*, the network-based solution is exactly the same as the one-sided version of Fisher’s exact test. In this sense, we generalize a standard functional enrichment approach for gene sets to networks induced by sets of genes.

### Network structure randomization

The function randomization approach for computing network-based functional enrichment tests the dependency of *cs*(*H_f_*) on the size of the set of genes annotated with *f*. However, *H_f_* also depends on the specific interactions present in the underlying universal network *G* = (*U*, *I*). To test the dependence of *cs*(*H_f_*) on *G*, we developed a separate method that relies on randomizing the structure of *G*. We use the following algorithm [17] to generate a randomized universal network with the same degree sequence as *G*, i.e., the degree of each node is the same in *G* and in the new network. This algorithm preserves topological properties of molecular interaction networks, such as their scale-free degree distribution.

The first *if* statement in RANDOMIZE prevents the algorithm from producing self-loops in the random network. The second *if* statement ensures that the edges to be added do not already exist in *I*′, thereby keeping the resulting graph simple. We perform the edge swap randomization for *k*|*I*| iterations, where *k* is an external parameter to this algorithm. We set *k* = 10 for all analysis presented in this paper. We determined this value for *k* by running RANDOMIZE with multiple values of *k* on the networks discussed in “Results”. For each value of *k*, we analyzed the distribution of the overlap between the edges in a random network and the original network. Increasing *k* beyond 10 did not show any significant change in this distribution (compared to *k* = 10) for any of the original networks.

In order to assess the significance of *cs*(*H_f_*) for each function, we repeatedly generate a randomized universal network *G′* = RANDOMIZE(*G*, *k*). Let *H′* = (*C*, *J′*) be the subgraph induced in *G′* by the genes in *C*, and let  be the subgraph of *H′* induced by the genes annotated with function *f*; *H′* and  are subgraphs of *G′* in the same fashion as *H* and *H_f_* are subgraphs of *G*. We calculate the *p-*value for each function *f* as the fraction of iterations (i.e., random networks) for which . This *p*-value indicates the probability that the genes in *H_f_* are more connected after randomly shuffling the edges in *G* (including those in *H_f_*) while maintaining the degree sequence of *G*.

### Combining *p*-values

We developed the function randomization approach because of its strong ties to the standard way we interpret functional enrichment. Furthermore, it can be expressed as an extension of the one-sided version of Fisher’s exact test. This approach is an attempt to answer the following question: “Given a function *f* that annotates *u_f_* genes, what is the probability that a randomly-selected set of *u_f_* genes will have a more connected intersection with the interesting genes than *H_f_* does?”.

As an alternative, we developed the network structure randomization approach because we were interested in a strictly network-based formulation of functional enrichment. This approach is an attempt to answer a very different question from the previous method: “Given a function *f*, what is the probability that the subgraph induced by genes in *C_f_* will be more connected in a network chosen uniformly at random from the set of all networks with the same degree sequence as *G*?”.

In this paper, we only consider those functions deemed well-connected by *both* approaches. Thus, we assign a *p*-value for each function that is the maximum of the two *p*-values from each of the two approaches. In the rest of the paper, unless stated otherwise, the *p*-value for a function refers to the maximum of the *p*-values computed by both approaches.

## Results

We present applications of our methods to three different interaction networks, each for a different organism. In the first application, we identify biological processes from the Gene Ontology (GO) [18] with significant network-based enrichment in the human B cell interactome [19]. In the second application, we discuss gene sets from the Molecular Signatures Database [20] that are deemed significantly enriched in a response network that represents the differences between collagen sandwiches and hepatocyte monolayers, two widely-used systems to culture rat hepatocytes [21]. In the third application, we discover GO biological processes whose network-based enrichment improves when we add a collection of genetic interactions in *S. cerevisiae* that were identified in a large-scale study conducted by Krogan *et al*. [22] to the BioGRID network [23].

Each of these three applications of network-based enrichment showcases how our methods can be used in a different manner: *i*) determine which functions are enriched in a given network, *ii*) given a network and an “interesting” sub-network of genes within that network, determine which functions are enriched in the sub-network, and *iii*) given two networks, determine the functions whose connectivity improves by merging the second network into the first. These three applications ask different questions about the functional enrichment of an interaction network, each of which we can answer using the framework presented in “Methods”.

We used the Synergizer [24] to generate all gene and protein identifier mappings. We visualized all networks using Cytoscape [25].

### B cell interactome

To analyze functions enriched in human B cells, we applied our methods to the B cell interactome (BCI) [19], which incorporates three different types of interactions: protein-protein, protein-DNA, and transcription factor-modulator. The BCI includes both experimentally verified and computationally predicted interactions [12,13] in human B cells. Protein-DNA and transcription factor-modular interactions are directed. For the purposes of this work, we treated them as being undirected. We removed repeated edges and self-loops from the BCI, resulting in an interaction network with 5748 nodes and 64,007 edges. We applied our network-based enrichment methods to identify GO biological processes enriched in the BCI. After downloading functional annotations from the GO website [18], we applied the true-path rule to the annotations, i.e., if a gene was annotated with function *f*, we ensured that it was also annotated with any ancestors of *f* in the GO directed acyclic graph.

Recall that our enrichment methods require a universal network *G* and an interesting network *H*. Since we were simply interested in the set of functions enriched in the BCI, we let both the universal network and the interesting network be the BCI. Notice that the standard formulation of gene set enrichment (Equation 1) will assign a *p*-value of 1 to every function, since the size of the interesting set of genes and the size of the universe are equal (i.e., *u* = *c* and *u_f_* = *c_f_*). As a result, we cannot compare our method for discovering enriched functions to the set-based Fisher’s exact test for this application. However, our approach assigns non-trivial *p*-values to several relevant functions, as we show below.

**Figure 2:**
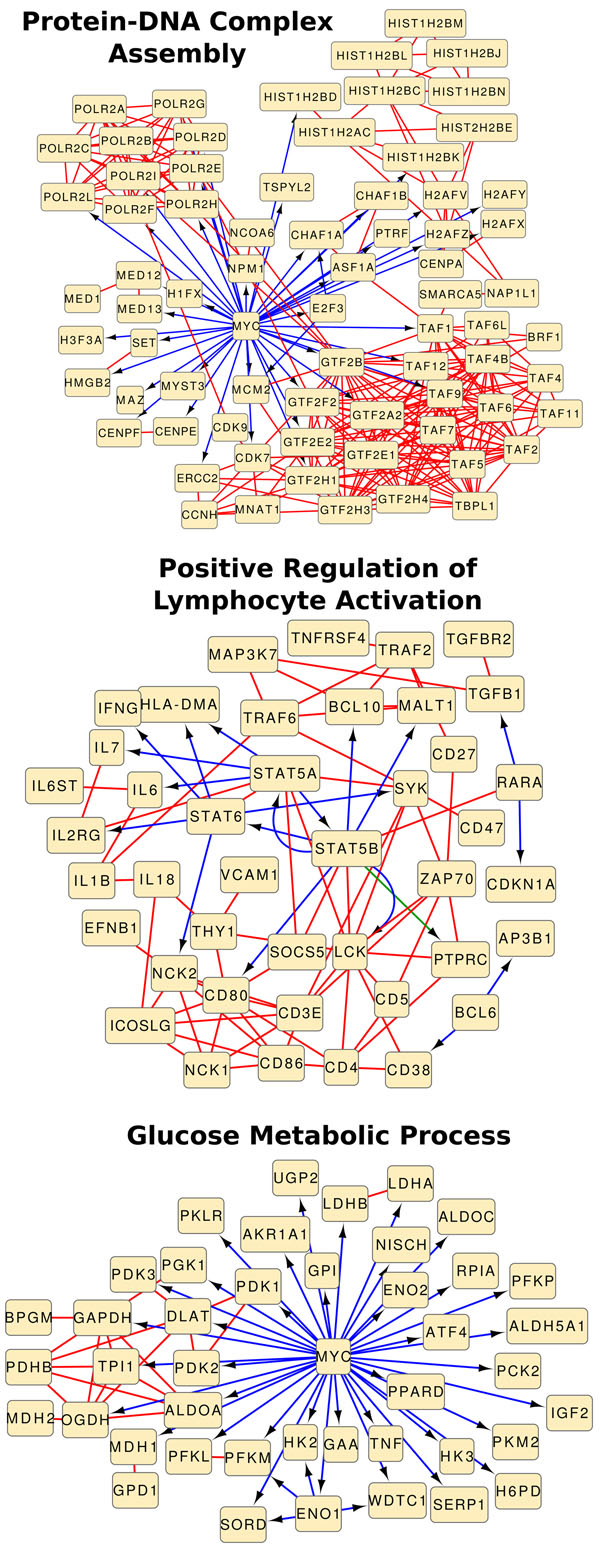
**Functions related to B cells**. Subgraphs of the BCI induced by genes annotated with (Top) GO:0065004 Protein-DNA Complex Assembly, (Middle) GO:0051251 Positive Regulation of Lymphocyte Activation, and (Bottom) GO:0006006 Glucose Metabolic Process. The red, blue, and green edges represent protein-protein, protein-DNA, and transcription factor-modulator interactions, respectively.

We computed *p*-values by executing each random sampling approach 100,000 times. We retained those functions that annotated at least five genes in the universal network and no more than 100 genes in the interesting network, resulting in a collection of 2098 GO biological processes. Of these, 31 processes received a network-based enrichment *p*-value of less than 10^–5^, which is the smallest empirical *p*-value possible over 100,000 iterations. Next, we present three significantly-enriched functions and discuss their relationship to the B cell interactome. The goal of this analysis is to demonstrate that network-based enrichment methods were capable of finding meaningful functions in a network associated with a specific cellular context. Figure 2 illustrates the subnetwork induced by the genes annotated with each of the three functions. In other words, each of the networks in Figure 2 is a visualization of the subgraph *H_f_* corresponding to one of three discussed functions. These figures only retain nodes that are incident on at least one edge. While we do not incorporate the directionality of the protein-DNA and transcription factor-modulator interactions in our methods, we considered the directionality when visualizing the results. We discuss functions that were ranked 1, 32, and 43 overall. Note that 31 processes received the smallest *p*-values. Hence, all of them have the rank of 1. We observed that a majority of these functions were related to protein phosphorylation and kinase signaling cascades. The term we discuss next is one that we could relate to B-cells based on the proteins annotated by that term.

#### Protein-DNA complex assembly

The GO term “Protein-DNA Complex Assembly” (GO:0065004) was enriched in the BCI with a *p*-value of less than 10^–5^ (rank 1 out of 2098 functions). Figure 2 demonstrates that the genes annotated by this function form several densely-connected subgraphs. A clustering algorithm may have detected each individual subgraph but may not have included all of them in a single cluster. Thus, the term “Protein-DNA Complex Assembly” may be determined to be enriched (using set-based approaches) in the results of a clustering algorithm only if the clusters were themselves further grouped during a post-processing procedure.

The genes in the subgraph induced by this function are associated with various aspects of the aggregation and binding of proteins with DNA molecules to form a protein-DNA complex. For example, the nine HIST and four H2A proteins are grouped together. DNA wraps around the histones, forming higher-order complex protein-DNA subunits. Many eukaryotic genes contain a DNA signature in their promoter region known as the TATA box. These genes rely on the ordered assembly of RNA polymerase II and several initiation factors at the TATA box. The ten POLR genes that form a clique encode different subunits of RNA polymerase II. The eight densely-connected general transcription factors (GTF2 genes in Figure 2) serve as initiation factors for RNA polymerase II. The eleven neighboring TAF genes perform a similar role. This function was likely determined to be enriched because of the hub transcription factor MYC, which serves as a bridge between these densely-connected sub-networks. Since MYC is responsible for regulating many other genes, irregular expression of MYC has been linked to several cancers, and some studies have identified this gene as a potential cancer drug target in humans [26].

#### Lymphocyte activation

We found the term “Positive Regulation of Lymphocyte Activation” (GO:0051251) enriched in the BCI with a *p*-value of 10^–5^ (rank 33). We expected to discover this function as enriched in the BCI: since B cells are a specific type of lymphocyte, up-regulation of lymphocyte activation is an inherent property of genes in the BCI. Our methods were capable of determining not only that many genes related to lymphocyte regulation appear in the BCI, but that there was a rich interconnectivity among these genes. Figure 2 illustrates that the signal transducer and activator of transcription (STAT) proteins (STAT5A, STAT5B, STAT6) are central to this connectivity. STAT5 has been shown to play a key role in the development and proliferation of B cells [27,28] through its activation by and mediation of many interleukins (e.g., IL2, IL6, and IL7, which are also present in this network). Each of these interleukins serves as a growth factor for various B cell lineages.

#### Glucose metabolism

We found the term “Glucose Metabolic Process” (GO:0006006) enriched in the BCI with a *p*-value of 2 × 10^–5^ (rank 43). While glucose metabolism is not solely related to the behavior of B cells, this process is important for many cell types, including B cells, as a primary source of energy. Perhaps what was most interesting about this function was its reliance on MYC for most of its connectivity. As mentioned, MYC is considered to be a master regulator because it regulates the activity of a large number of human genes. Figure 2 demonstrates that without the annotation of MYC by “Glucose Metabolic Process” the network would consist of mostly disconnected proteins. While MYC also plays a central role in “Protein-DNA Complex Assembly”, one could argue that, even without MYC and the edges incident on it, the proten-DNA complex assembly network is reasonably well-connected and will be deemed to be significantly enriched by our methods. In the case of “Glucose Metabolic Process,” we elucidated a function whose network-based enrichment score relies heavily on its connectivity through a central hub.

### Hepatic cultures

The liver carries out a multitude of necessary functions in humans and many other animals, including the metabolism of foreign compounds (xenobiotics) and cholesterol. Hepatocytes constitute roughly 70-80% of the liver cells. Two commonly used systems for culturing these cells *in vitro* are the hepatocyte monolayer (HM) and the collagen sandwich (CS). Briefly, the HM consists of a layer of collagen on top of which a single layer of hepatocytes are placed; the CS is similar to the HM with the addition of an extra layer of collagen on top (creating a “sandwich” of hepatocytes between two layers of collagen). A recent study analyzed the expression profile over eight days of genes in primary rat hepatocytes in both HM and CS tissue cultures [21]. The authors demonstrated that over eight days, the genes annotated by a wide range of liver-specific biological processes were consistently up-regulated in hepatocytes in CS but not in HM and that processes related to the cell cycle were down-regulated in CS, as compared to HM. Hepatocytes in HM differentiate and lose some of their morphology over time, contributing to their loss of liver-specific functions.

For this application of our enrichment methods, we sought to discover functions that annotate differentially-expressed genes in the CS cultures when compared to HMs. We used the STRING database of known and predicted protein interations [29] as our universal interaction network. STRING assigns a weight between 0 and 1000 to each interaction based on the combined scores of various sources of evidence for the interaction, where higher scores indicate more confidence for that interaction. We removed any self-loops or interactions with a score below 500 from the universal network, resulting in a network of 9925 nodes and 204,992 edges. We analyzed the expression profiles for rat hepatocyte genes in HM versus CS cultures after 8 days of growth [21], since CS showed the greatest divergence from HM after 8 days. Based on these expression profiles, we used LIMMA [30] to compute *p*-values that represented the significance of the differential expression of each gene between CS and HM. We desired to compute the response network (a subgraph of the STRING network) that captured the differential expression between CS and HM cultures. Accordingly, we used these per-gene *p*-values in concert with the BioNet algorithm [31] to compute a response network (within the STRING universal network) of interconnected genes that is perturbed in CS, in comparison to HM. We used the default parameters for BioNet, with FDR=0.001. Please see the BioNet publication for more details. The response network contained 876 nodes and 3423 edges. We collected functional annotations of the genes in this network from the Molecular Signatures Database (MSigDB) [20]. We only retained curated gene sets (category C2) and GO gene sets (category C5) from MSigDB, and we removed any functions that annotated fewer than five genes in the universal interaction network.

**Figure 3:**
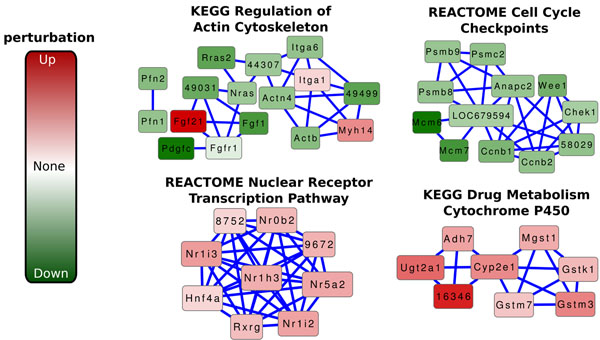
**Functions related to hepatic cultures.**Subgraphs of a hepatocyte response network induced by genes annotated with MSigDB gene sets. (Top Left) (KEGG) Regulation of Actin Cytoskeleton, (Top Right) (REACTOME) Cell Cycle Checkpoints, (Bottom Left) (REACTOME) Nuclear Receptor Transcription Pathway, and (Bottom Right) (KEGG) Drug Metabolism Cytochrome P450. Red and green nodes indicate up- and down-regulation, respectively, of individual genes in collagen sandwich versus hepatocyte monolayer tissue cultures. Darker node color indicates higher perturbation in either direction, as indicated by the legend on the left.

With the universal interaction network, an interesting subnetwork of the universe (i.e., the BioNet response network), and functional annotations of the universal genes, we applied our network-based enrichment methods. We tested the enrichment of 4035 MSigDB functions for 100,000 iterations. We also computed the enrichment of each function, using an empirical version of Equation 1, to allow us to compare our network-based enrichment *p*-value for a given function to the *p*-value given by a standard gene set-based enrichment method (i.e., the one-sided version of Fisher’s exact test). We did not use the analytical version of Fisher’s test so that the enrichment *p*-value estimated by our methods for each function was comparable to that computed by Fisher’s test. We observed that 177 of the 4035 functions received a network-based enrichment *p*-value of less than 10^–5^, the smallest possible empirical *p*-value for 100,000 iterations (i.e., our methods returned a *p*-value of 0 for these functions). We present four of the top 177 functions and analyze their relationship to liver cultures. Figure 3 illustrates the networks induced by the genes annotated by these four top-ranking network-based enriched functions (i.e., we show *H_f_* for each function). The genes are colored based on their level of perturbation in the contrast between CS and HM. Lightly-colored genes indicate little or no perturbation, while dark red (green) nodes denote significant up- (down-) regulation in CS compared to HM.

#### Actin cytoskeleton

Our network-based enrichment methods discovered the KEGG [3] pathway “Regulation of Actin Cytoskeleton” enriched with a *p*-value less than 10^–5^ (rank 1). Comparatively, the empirical *p*-value for the one-sided version of Fisher’s exact test was 0.02857 (rank 819), more than three orders of magnitude larger than our *p*-value. Actin organizes into thin filaments to provide structure to cells. Cell locomotion is often a driving force for the development of actin cytoskeleton. Actin filaments form a highly-organized, complex structure, and manipulation of the actin cytoskeleton enables adhesion to the subtrate and movement of the cell [32]. However, hepatocytes are generally stationary cells and are unlikely to use this mechanism. More specifically, HM hepatocytes lose the true hepatic phenotypes much faster than those in CS cultures. A specific characteristic of this loss is the production of actin fibers that help cells in the HM culture to better adhere to the underlying substrate. This phenomenon explains why many genes in the “Regulation of Actin Cytoskeleton” network (Figure 3) are consistently down-regulated in CS hepatocytes compared to those in HM. Note that this network contains some up-regulated genes, including Fgf21 and Myh14. These genes are involved in a wide variety of biological processes including cell growth, tissue repair, and cell polarity. Their participation in several processes not directly related to actin development may explain why they are upregulated in CS compared to HM in this network.

#### Cell cycle checkpoints

We found the REACTOME [1] pathway “Cell Cycle Checkpoints” enriched with a *p*-value less than 10^–5^ (rank 1), while the empirical version of Fisher’s method assigned a *p*-value of 0.05539 (rank 1034). Many genes in this network are essential to the cell cycle process. ANAPC2 codes for part of the anaphase-promoting complex, which is responsible for promoting eukaryotic cells from metaphase to anaphase during mitosis. Wee1 serves as a key checkpoint in the cell cycle by inhibiting entry into mitosis until the cell has grown to a certain size, thus preventing the separation of the cell into two daughter cells that are too small. Mcm6 and Mcm7 are essential genes for DNA replication in eukaryotes, a significant process in the cell cycle. Under healthy conditions, mature hepatocytes do not readily divide except to replace damaged hepatocytes [33]. This observation may explain the downregulation of these genes in CS compared to HM. In other words, down-regulation of cell cycle checkpoints prevents the cell cycle from progressing in CS cultures, in comparison to HM cultures.

#### Nuclear receptor transcription pathway

We identified the REACTOME “Nuclear Receptor Transcription Pathway” enriched with a network-based *p*-value less than 10^–5^ (rank 1). Fisher’s method returned a *p*-value of 0.0092 (rank 565). Nuclear receptors are responsible for sensing steroids, hormones, and other signaling molecules in the cell. Since one of the primary functions of the liver is the production of hormones, enrichment of this gene set in CS cultures is to be expected and was noted in an earlier study [21]. Furthermore, we expect many genes involved in this pathway to exhibit up-regulation in CS versus HM cultures. Figure 3 confirms that all the genes in the intersection between this pathway and the BioNet response network are indeed up-regulated. Nr1h3, Nr1i2, Rxrg, and Hnf4a encode parts of the liver X receptor, the pregnane X receptor, the retinoic acid receptor, and the hepatocyte nuclear factor, respectively. All these nuclear receptors are responsible for sensing signaling molecules that are highly relevant to liver function, e.g., metabolism of toxic substances and vitamins.

#### Drug metabolism

The KEGG pathway “Drug Metabolism of Cytochrome P450” was enriched according to our network-based method with a *p*-value of less than 10^–5^ (rank 1). Fisher’s method assigned the same function a *p*-value of 0.01572 (rank 681). All genes in this network were up-regulated in CS hepatocytes compared to those in HM. One of the primary functions of the liver is processing xenobiotics, including drugs. Cytochrome P450s represent a class of proteins that are responsible for breaking down various lipids, steroids, and xenobiotic (external) compounds. Primary hepatocytes actively express these proteins, even in the absence of external chemicals or drugs [34]. The enrichment of this pathway in CS cultures (in comparison to HMs) supports the well-known phenomenon that HMs rapidly lose liver-specific functions.

#### Gene sets with high network-based *p*-values

Several functions received a high network-based *p*-value from our methods and a low set-based *p*-value using Fisher’s exact test. We ranked the genes sets in increasing order of their set-based *p*-values, and we report the two highest-ranking gene sets that received a network-based *p*-value of 1. According to Fisher’s exact test, we found the MSigDB gene set “Su Liver” significantly enriched with a *p*-value of 1.77^–6^ (rank 89) and the gene set “Chiaradonna Neoplastic Transformation Kras DN” significantly enriched with a *p*-value of 2.48 × 10^–6^ (rank 93). Our network-based functional enrichment approach deemed both functions insignificant with a *p*-value of 1 because there exist no interactions among the genes annotated by either function. Gene sets whose constituent proteins are sparsely-connected may be difficult to interpret.

### Improving functional network coherence

In the third and final application of our network-based enrichment methods, we sought to analyze how the addition of a collection of experimentally determined interactions impacts a dataset of known interactions. Specifically, given a universal interaction network in *S. cerevisiae*, we wanted to determine which biological functions became more coherent within the network after introducing new pairwise interactions discovered in a large-scale genetic interaction (GI) study. We retrieved the yeast interactome from the BioGRID database [23], which incorporates both genetic and physical interactions from multiple sources (5681 nodes and 97,862 edges). BioGRID includes a sub-network of 14,421 GIs among 721 genes reported by Collins et al [22]. We refer to this list of GIs as “the GI study” or “the GIs” throughout this section. Our goal was to ask which GO biological processes became better connected when the GIs were added to BioGRID. We downloaded GO biological process annotations of the genes in BioGRID from GO [18]. As with the annotations of the BCI, we applied the true-path rule to the annotations, and we removed any functions that annotated fewer than five genes in the universal network and more than 100 genes in the interesting collection *C*.

We then applied our network-based enrichment approach in two ways. The universal interaction network (*G*) remained the same in both cases. In the first case, we set the interesting network (*H*) to be the same as the universal network (which also includes edges from the GI study). This analysis is similar to the analysis performed on the BCI. Essentially, the network-based *p*-values in this case indicated which functions were enriched in the entire BioGRID network, including the GIs. For the second case, we set the interesting network to be the universal network with interactions from the GI study removed. If there were other sources of evidence for one of the GIs, we retained that edge in the interesting network. The network-based *p*-values in this case indicated which functions were enriched in the universal network if we ignored any information from the GI study.

Let *p_a_*(*f*) be the network-based *p*-value for function *f* from the first case, where we considered all edges in the univeral network, including those from the GI study. Similarly, let *p_r_*(*f*) be the network-based *p*-value for function *f* from the second case, where we removed any edges in the GI study from the interesting network. We scored each function by *s*(*f*) = log_10_(*p_r_*(*f*)/*p_a_*(*f*)). Thus, functions whose network-based enrichment *p*-value decreased (became more significant) as a result of adding the GIs received a positive score, functions whose enrichment remained the same received a score of 0, and functions whose *p*-values increased (became less significant) as a result of adding the GIs received a negative score. We believed that highly positive-scoring functions were those biological processes whose coherence improved the most by adding the GIs. Of the 1443 functions tested, 28 functions received a score greater than 1 (i.e., the *p*-value decreased by more than 1 order of magnitude after adding the GIs), and only 1 function received a score less than -1 (i.e., the *p*-value increased by more than 1 order of magnitude by including the GIs). Next, we discuss the three GO biological processes, “Chronological Cell Aging”, “DNA Geometric Change”, and “Histone Deacetylation”, that received the 2nd, 3rd, and 9th highest overall scores, respectively. These examples illustrate a new application of network-based functional enrichment that is sensitive to the increased connectivity among a set of genes upon the addition of multiple edges. However, gene set-based enrichment may not be sensitive to such an addition of edges if, for example, all newly-added edges were among genes already present in the network.

#### Cell aging

**Figure 4:**
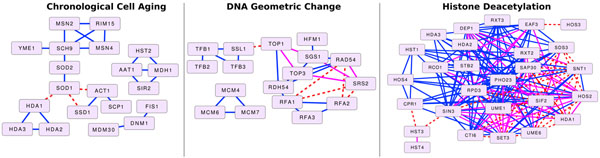
**Functions related to the GI study**. Subgraphs of the BioGRID universal network induced by genes annotated with GO biological processes (Left) GO:0001300 Chronological Cell Aging, (Middle) GO:0032392 DNA Geometric Change, and (Right) GO:0016575 Histone Deacetylation. Blue interactions are edges in BioGRID that were not identified by the GI study [22]. Solid pink edges were identified in the GI study and were also present in BioGRID through some alternative evidence. Dashed red interactions were only discovered by the GI study.

The GO biological process “Chronological Cell Aging” (GO:0001300) received a score of 2.6464 (*p_a_* = 0.0003 and *p_r_* = 0.1329) exhibiting a decrease in network-based *p*-value of nearly three orders of magnitude with the addition of the GIs. This process is the progression of quiescent (non-dividing) cells from their inception to the end of their lifespans. Figure 4 demonstrates that this increase in enrichment is a result of three GIs incident on SOD1, a Cu-Zn superoxide dismutase that has a role in the detoxification of oxygen radicals. These enzymes catalyze the breakdown of the superoxide radical (O2-) into an oxygen molecule and hydrogen peroxide. Yeast cultures lacking SOD1 exhibit drastically decreased viability [35]. Strikingly, the interactions from the GI study were able to connect three previously disconnected subnetworks of genes annotated with chronological cell aging in *S. Cerevisiae*.

#### DNA geometric change

Our approach identified the GO term “DNA Geometric Change” (GO:0032392) with a score of 2.32 as having a considerably decreased network-based enrichment *p*-value after adding the GIs (*p_a_* less than 10^–4^, but *p_r_* = 0.0021). The *p*-value decreased primarily because of the GI between SSL1 and TOP1, which connected two previously disconnected components of the network induced by genes associated with DNA geometric change. Additionally, the added GIs reinforced the connectivity among RFA1, RFA2, RAD54, SRS2, and their neighboring genes. Thus, the connected component induced by these genes was less likely to become disconnected during the randomization procedures used to perform network-based enrichment.

#### Histone deacetylation

Our enrichment approach assigned a score of 1.5955 (*p_a_* = 0.0005 and *p_r_* = 0.0197) to the GO term “Histone Deacetylation” (GO:0016575). The GIs improved the connectivity among several genes related to histone deacetylation (HDA1, HOS2, SDS3, SIF2, SNT1, UME1, and UME6). The GIs also provided a new source of evidence for many previously-known interactions, a property we can deduce from Figure 4 but one that is not explicitly considered by our methods. However, the interaction connecting HOS3 to EAF3 and the interactions between HST3 and CPR1/SIN3 are perhaps the most interesting as they provide new evidence for including HOS3, HST3, and HST4 in the large connected component associated with histone deacetylation. The GIs that connect HST3 and HST4 to the largest connected component of this biological process are quite striking, since these genes code for histone deacetylases, and the GIs provide previously unknown interaction data for including them in this network.

## Conclusions

In this paper, we have presented a novel approach to functional enrichment that takes into account the pairwise relationships among genes annotated by a particular function. We proposed two different methods for calculating *p*-values to assess the significance of each function in a network-based context; we described these methods as “function randomization” and “network structure randomization”. We required functions to have low enrichment *p*-values based on both criteria. Our function randomization approach is a generalization of the one-sided version of Fisher’s exact test, a standard formulation of functional enrichment for gene sets. Specifically, after removing edges from the universal and interesting networks, the function randomization approach is equivalent to the one-sided version of Fisher’s exact test. Our network structure randomization approach offers a strictly network-centric method for determining functional enrichment.

We utilized our methods on real biological data from three different organisms: human, rat, and yeast. In each organism, we showcased a different application of network-based enrichment. First, we used the human B cell interactome to demonstrate the capability of our methods to simply discover enriched functions in a single network. We have noted that many standard gene set enrichment methods do not address this issue, as they require a universal gene set and an interesting collection of genes within the universal genes. Moreover, graph clustering algorithms that are designed to compute dense subgraphs might not detect sub-networks that are well-connected but not very dense, such as those corresponding to “Protein-DNA Complex Assembly” and “Glucose Metabolic Process” in Figure 2. Second, we applied our methods to a response network composed of rat genes perturbed in hepatocytes cultured in two different ways. This approach parallels traditional gene set enrichment methods, where given an interesting collection of genes, we seek functions overrepresented in the interesting genes with respect to some universe of genes. We identify several relevant functions that our network-based method finds as being most significant but are far from the highest-ranking functions using the set-based Fisher’s method. Third, we demonstrated a novel application of network-based enrichment to assess the functional contribution of a collection of genetic interactions in yeast to the underlying universe of known yeast interactions. 

Our work suggests many directions of future research. While we did not take edge reliabilities into account for the analysis presented here, our methods can be readily applied to weighted networks. Indeed, our approach for comparing sequences of component sizes can be used to compare sorted sequences of the sums of edge weights within each component (rather than the number of nodes). Identifying alternative methods for sequence comparison is also an interesting question, particularly those methods for which the resulting distribution of sorted sequences can be determined analytically. Such comparison methods may drastically improve the computational efficiency of our network-based enrichment approach. Our methods analyze the functions one at a time and often lead to redundant functions being identified as enriched. Additionally, we need to apply corrections for multiple hypotheses testing, thereby potentially decreasing our statistical power. Furthermore, our permutation-based approach for computing *p*-values is very time-consuming. These three issues can potentially be tackled by generalizing model-based approaches for gene function enrichment [15,16] to the domain of network-based function enrichment. After assuming an appropriate model for how biological processes may be perturbed in a cell, these methods proceed to infer the set of biological processes that best explain an input list of genes (the interesting collection) against a background list (the universe). We are developing a modified version of our network-based approach that identifies groups of functions that jointly cover the network of interest, rather than consider functions individually. Applying these methods in the network context is an interesting and important problem.

## Competing interests

The authors declare that they have no competing interests.

## Authors’ contributions

All authors aided in the design of the methods. TMM conceived the idea and contributed to implementation of the methods. CLP and CCO implemented the algorithms. CLP collected the data and performed the analysis. CLP and TMM interpreted the results and wrote the manuscript.

## References

[B1] Joshi-Tope G Gillespie M Vastrik I D’Eustachio P Schmidt E de Bono B Jassal B Gopinath G Wu G Matthews L Lewis S Birney E Stein L Reactome: a knowledgebase of biological pathways Nucleic Acids Res 2005 33 Database issue D428 32 15608231 10.1093/nar/gki072 PMC540026

[B2] Reguly T Breitkreutz A Boucher L Breitkreutz BJ Hon GC Myers CL Parsons A Friesen H Oughtred R Tong A Stark C Ho Y Botstein D Andrews B Boone C Troyanskya OG Ideker T Dolinski K Batada NN Tyers M Comprehensive curation and analysis of global interaction networks in Saccharomyces cerevisiae Journal of Biology 2006 5 4 11+ 10.1186/jbiol36 16762047 PMC1561585

[B3] Kanehisa M Goto S Hattori M Aoki-Kinoshita KF Itoh M Kawashima S Katayama T Araki M Hirakawa M From genomics to chemical genomics: new developments in KEGG Nucleic Acids Res 2006 34 Database issue D354 7 16381885 10.1093/nar/gkj102 PMC1347464

[B4] Rual J Towards a proteome-scale map of the human protein-protein interaction network Nature 2005 437 7062 1173 8 10.1038/nature04209 16189514

[B5] Uetz P Giot L Cagney G Mansfield TA A comprehensive analysis of protein-protein interactions in Saccharomyces cerevisiae Nature 2000 403 6770 623 627 10.1038/35001009 10688190

[B6] Harbison C Gordon D Lee T Rinaldi N Transcriptional regulatory code of a eukaryotic genome Nature 2004 431 7004 99 104 10.1038/nature02800 15343339 PMC3006441

[B7] arkowetz F Spang R Inferring cellular networks - a review BMC Bioinformatics 2007 8 Suppl 6 10.1186/1471-2105-8-S6-S5 PMC1995541 17903286

[B8] Bansal M Belcastro V Ambesi-Impiombato A di Bernardo D How to infer gene networks from expression profiles Mol Syst Biol 2007 3 10.1038/msb4100120 PMC1828749 17299415

[B9] Ideker T Ozier O Schwikowski B Siegel AF Discovering regulatory and signalling circuits in molecular interaction networks Bioinformatics 2002 18 Suppl 1 S233 40 10.1093/bioinformatics/18.suppl_1.S233 12169552

[B10] Dittrich MT Klau GW Rosenwald A Dandekar T Muller T Identifying functional modules in protein-protein interaction networks: an integrated exact approach Bioinformatics 2008 24 13 i223 231 10.1093/bioinformatics/btn161 18586718 PMC2718639

[B11] Ulitsky I Shamir R Identification of functional modules using network topology and high-throughput data BMC Syst Biol 2007 1 8 10.1186/1752-0509-1-8 17408515 PMC1839897

[B12] Margolin AA Wang K Lim WK Kustagi M Nemenman I Califano A Reverse engineering cellular networks Nature Protocols 2006 1 2 662 671 10.1038/nprot.2006.106 17406294

[B13] Wang K Saito M Bisikirska BC Alvarez MJ Lim WK Rajbhandari P Shen Q Nemenman I Basso K Margolin AA Klein U Dalla-Favera R Califano A Genome-wide identification of post-translational modulators of transcription factor activity in human B cells Nature Biotechnology 2009 27 9 829 837 10.1038/nbt.1563 19741643 PMC2753889

[B14] Grossmann S Bauer S Robinson PN Vingron M Improved detection of overrepresentation of gene-ontology annotations with parent-child analysis Bioinformatics 2007 10.1093/bioinformatics/btm440 17848398

[B15] Bauer S Gagneur J Robinson PN GOing Bayesian: model-based gene set analysis of genome-scale data Nucleic acids research 2010 38 11 3523 3532 10.1093/nar/gkq045 20172960 PMC2887944

[B16] Lu Y Rosenfeld R Simon I Nau GJ Bar-Joseph Z A probabilistic generative model for GO enrichment analysis Nucl. Acids Res 2008 36 17 e109+ 18676451 10.1093/nar/gkn434 PMC2553574

[B17] Milo R Kashtan N Itzkovitz S Newman M Alon U On the uniform generation of random graphs with prescribed degree sequences Arxiv preprint cond-mat/0312028 2003

[B18] Ashburner M Ball CA Blake JA Botstein D Butler H Cherry JM Davis AP Dolinski K Dwight SS Eppig JT Harris MA Hill DP Kasarskis A Lewis S Matese JC Richardson JE Ringwald M Rubin GM Sherlock G Gene Ontology: tool for the unification of biology. The Gene Ontology Consortium Nat Genet 2000 25 25 9 10.1038/75556 10802651 PMC3037419

[B19] Lefebvre C Lim WK Basso K Favera RD Califano A A context-specific network of protein-DNA and protein-protein interactions reveals new regulatory motifs in human B cells Proceedings of the Joint 2006 satellite conference on Systems Biology and Computational Proteomics 2007 Berlin, Heidelberg: Springer-Verlag 42 56

[B20] Subramanian A Tamayo P Mootha V Mukherjee S Ebert B Gillette M Paulovich A Pomeroy S Golub T Lander E Mesirov J Gene set enrichment analysis: a knowledge-based approach for interpreting genome-wide expression profiles Proc Natl Acad Sci U S A 2005 10.1073/pnas.0506580102 PMC1239896 16199517

[B21] Kim Y Lasher CD Milford LM Murali TM Rajagopalan P A comparative study of genome-wide transcriptional profiles of primary hepatocytes in collagen sandwich and monolayer cultures Tissue Eng Part C Methods 2010 10.1089/ten.tec.2010.0012 PMC2988646 20412007

[B22] Collins SR Miller KM Maas NL Roguev A Fillingham J Chu CS Schuldiner M Gebbia M Recht J Shales M Ding H Xu H Han J Ingvarsdottir K Cheng B Andrews B Boone C Berger SL Hieter P Zhang Z Brown GW Ingles CJ Emili A Allis CD Toczyski DP Weissman JS Greenblatt JF Krogan NJ Functional dissection of protein complexes involved in yeast chromosome biology using a genetic interaction map Nature 2007 446 7137 806 810 10.1038/nature05649 17314980

[B23] Stark C Breitkreutz BJJ Chatr-Aryamontri A Boucher L Oughtred R Livstone MS Nixon J Van Auken K Wang X Shi X Reguly T Rust JM Winter A Dolinski K Tyers M The BioGRID Interaction Database: 2011 update Nucleic Acids Research 2011 39 Database issue D698 D704 21071413 10.1093/nar/gkq1116 PMC3013707

[B24] Berriz GF Roth FP The Synergizer service for translating gene, protein and other biological identifiers Bioinformatics 2008 24 19 2272 2273 10.1093/bioinformatics/btn424 18697767 PMC2553440

[B25] Shannon P Markiel A Ozier O Baliga NS Wang JT Ramage D Amin N Schwikowski B Ideker T Cytoscape: a software environment for integrated models of biomolecular interaction networks Genome Res 2003 13 11 2498 504 10.1101/gr.1239303 14597658 PMC403769

[B26] Soucek L Whitfield J Martins CP Finch AJ Murphy DJ Sodir NM Karnezis AN Swigart LB Nasi S Evan GI Modelling Myc inhibition as a cancer therapy Nature 2008 455 7213 679 683 10.1038/nature07260 18716624 PMC4485609

[B27] Heltemes-Harris LM Willette MJL Vang KB Farrar MA The role of STAT5 in the development, function, and transformation of B and T lymphocytes Annals of the New York Academy of Sciences 2011 1217 18 31 10.1111/j.1749-6632.2010.05907.x 21276004

[B28] Malin S McManus S Busslinger M STAT5 in B cell development and leukemia Current Opinion in Immunology 2010 10.1016/j.coi.2010.02.004 20227268

[B29] Szklarczyk D Franceschini A Kuhn M Simonovic M Roth A Minguez P Doerks T Stark M Muller J Bork P Jensen LJ von Mering C The STRING database in 2011: functional interaction networks of proteins, globally integrated and scored Nucleic Acids Research 2011 39 Database issue D561 D568 21045058 10.1093/nar/gkq973 PMC3013807

[B30] Smyth GK Bioinformatics and computational biology solutions using R and bioconductorchap Limma: linear models for microarray data 2005 Springer 397 420

[B31] Beisser D Klau GW Dandekar T Muller T Dittrich MT BioNet: an R-Package for the functional analysis of biological networks Bioinformatics 2010 26 8 1129 30 10.1093/bioinformatics/btq089 20189939

[B32] Welf ES Haugh JM Signaling pathways that control cell migration: models and analysis Wiley interdisciplinary reviews. Systems biology and medicine 2011 3 2 231 240 10.1002/wsbm.110 21305705 PMC3052860

[B33] Mohammed FF Khokha R Thinking outside the cell: proteases regulate hepatocyte division Trends in Cell Biology 2005 15 10 555 563 10.1016/j.tcb.2005.08.009 16150595

[B34] Hewitt NJ Gómez Lechón MJ Houston JB Hallifax D Brown HS Maurel P Kenna JG Gustavsson L Lohmann C Skonberg C Guillouzo A Tuschl G Li AP LeCluyse E Groothuis GMM Hengstler JG Primary hepatocytes: current understanding of the regulation of metabolic enzymes and transporter proteins, and pharmaceutical practice for the use of hepatocytes in metabolism, enzyme induction, transporter, clearance, and hepatotoxicity studies Drug Metabolism Reviews 2007 39 159 234 10.1080/03602530601093489 17364884

[B35] Longo VD Gralla EB Valentine JS Superoxide dismutase activity is essential for stationary phase survival in Saccharomyces cerevisiae. Mitochondrial production of toxic oxygen species in vivo The Journal of Biological Chemistry 1996 271 21 12275 12280 10.1074/jbc.271.21.12275 8647826

